# Microbial Ecology along the Gastrointestinal Tract

**DOI:** 10.1264/jsme2.ME17017

**Published:** 2017-11-10

**Authors:** Ethan T. Hillman, Hang Lu, Tianming Yao, Cindy H. Nakatsu

**Affiliations:** 1 Department of Agricultural and Biological Engineering, Purdue University West Lafayette, Indiana 47907 USA; 2 Department of Animal Science, Purdue University West Lafayette, Indiana 47907 USA; 3 Department of Food Science, Purdue University West Lafayette, Indiana 47907 USA; 4 Department of Agronomy, Purdue University West Lafayette, Indiana 47907 USA

**Keywords:** Microbiome, mycobiome, virome, human gastrointestinal (GI) tract, animal models, diet

## Abstract

The ecosystem of the human gastrointestinal (GI) tract traverses a number of environmental, chemical, and physical conditions because it runs from the oral cavity to the anus. These differences in conditions along with food or other ingested substrates affect the composition and density of the microbiota as well as their functional roles by selecting those that are the most suitable for that environment. Previous studies have mostly focused on *Bacteria*, with the number of studies conducted on *Archaea*, *Eukarya*, and *Viruses* being limited despite their important roles in this ecosystem. Furthermore, due to the challenges associated with collecting samples directly from the inside of humans, many studies are still exploratory, with a primary focus on the composition of microbiomes. Thus, mechanistic studies to investigate functions are conducted using animal models. However, differences in physiology and microbiomes need to be clarified in order to aid in the translation of animal model findings into the context of humans. This review will highlight *Bacteria*, *Archaea*, *Fungi*, and *Viruses*, discuss differences along the GI tract of healthy humans, and perform comparisons with three common animal models: rats, mice, and pigs.

Researchers have been investigating the ecology of the intestinal microbiota for decades (120, 165) in order to identify, characterize, and count their numbers. These extensive efforts are due to the important roles the intestinal microbiota play in digestion, the production of essential vitamins, and protection of the gastrointestinal (GI) tract from pathogen colonization (141). In the past few decades, molecular techniques targeting the 16S rRNA gene and other genetic markers have been developed to characterize and analyze bacterial communities. These methods have been used to reveal the important roles played by microbes in the GI tract (23, 180, 183, 184, 189, 212). In healthy individuals, the microbiome (microbial community) and host have a mutualistic relationship in which both partners benefit; however, pathogens may invade and cause disease under certain conditions. The initial aim of most studies was to elucidate the role of the microbiome in disease. More recently, surveys have been performed on healthy individuals in order to assess the contribution of the microbiota to health, particularly in response to dietary changes/supplementation with probiotics and/or prebiotics.

The human GI tract is a complex system that starts from the oral cavity, continues through the stomach and intestines, and finally ends at the anus (Fig. 1). The density and composition of the microbiome change along the GI tract, with major populations being selected by the functions performed at the various locations. Bacteria along the GI tract have several possible functions, many of which are beneficial for health including vitamin production, the absorption of ions (Ca, Mg, and Fe), protection against pathogens, histological development, enhancement of the immune system, and the fermentation of “non-digestible foods” to short chain fatty acids (SCFA) and other metabolites (19, 58, 63, 77, 138). The roles of fungi and viruses have not been examined in as much detail; however, they are known to play important roles in microbiota dynamics and host physiology/immunity related to health and disease (45, 94, 133).

Food passes through the GI tract and the absorption rate of nutrients is largely dependent on the activities of various enzymes in the digestive system, such as amylase in saliva, pepsin in the stomach, and pancreatic enzymes in the small intestine. These mechanisms have been extensively examined (61, 62), particularly in the stomach. However, many food components cannot be digested in the upper GI tract and are passed into the lower intestinal tract, in which they are fermented by microbes. Functional studies commonly use animal models in order to obtain a better understanding of the processes in the GI tract that may lead to better health or decrease disease. However, information from animal models may not be directly translatable to humans. Therefore, researchers need to consider the limitations of the selected animal model when extrapolating findings to humans.

Although microbiome studies often include an ecological component, most of the research performed to date has focused on *Bacteria* and not all of the biota. This represents a logical approach because *Bacteria* comprise most of the microbiome. However, even biota representing a small proportion of the microbiome may play important roles in the ecosystem (133). Therefore, researchers need to start shifting their approach to include eukaryotic, prokaryotic, and viral (33, 133) interactions in efforts to elucidate the roles of all components of the microbiome.

In recent years, a number of reviews have summarized findings from the increasing number of studies being performed in this field (36, 73, 176, 188). While most studies have focused on disease, the microbiome is also important for maintaining health. We herein highlight differences in the microbiome (*Bacteria*, *Archaea*, *Fungi*, and *Viruses*) along the GI tract of healthy humans, and how it compares to those of typical animal models used in research. One finding that is consistent to most studies is that the microbiome of healthy individuals is unique; however, there are still some generalities that will be discussed in this review.

## Microbiome diversity

Many factors contribute to the diversity of microbiomes, and most studies have demonstrated the individuality of microbiomes among subjects. Previous findings support microbial communities being more similar in subjects that are genetically related (191), of a similar age (135, 213), or with common diets (including the influences of ethnicity and geography) (63). Diseases will also have an impact on microbiome diversity, including autoimmune and neoplastic diseases, such as inflammatory bowel disease, diabetes, obesity, cardiovascular diseases, allergies, and cancer (37, 121). Treatments for diseases may also affect a patient’s gut microbiota, and the consequences of antibiotic use have been intensively investigated (22, 95).

The host genotype has been shown to influence the development of the gut microbiota, and the immune system has been identified as a contributing factor (188). Crosstalk between the microbiome and human immune system occurs in response to a number of environmental factors, such as diet, xenobiotics, and pathogens. Microbial host interactions occur in the gut, mainly in the epithelial cell layer, myeloid cells, and innate lymphoid cells, in which crosstalk and feedback loops contribute to the microbiome composition, host physiology, and disease susceptibility. These interactions contribute not only to the bacterial community along the GI tract, but also to the other microbiota (*Fungi*, *Archaea*, and *Viruses*). Our understanding of the immunology associated with *Fungi* (150) and *Archaea* is currently limited. Transkingdom commensal relationships among microbiota (including *Viruses*) are considered to form from infancy (29, 30, 106, 200) and several co-occurring relationships have been identified (35, 75, 76, 85, 214).

### Bacteria

A more complete picture of human-associated bacterial communities obtained using molecular techniques has revealed that their diversity is greater than initially considered through cultivation (9, 20, 56, 90, 113). Using almost full-length 16S rRNA gene sequences, predicted taxa numbers range from 100–300 (20, 56), while pyrosequencing suggests there are 1000s of phylotypes (38, 49). Most of the gut bacteria identified by 16S rRNA gene sequencing belong to the five phyla originally identified by cultivation, namely, *Bacteroidetes*, *Firmicutes*, *Actinobacteria*, *Proteobacteria*, and *Verrucomicrobia* (90), and, at lower proportions, *Fusobacteria*, *Tenericutes*, *Spirochaetes*, *Cyanobacteria*, and TM7 (189). At lower levels of the taxonomic classification, microbiome compositions vary with each individual. Attempts have been made to identify a single core microbiome of *Bacteria* in the GI tract. Although this has not been possible in the lower GI tract (mainly using fecal samples) based on taxonomy, it appears there are core microbial functions (152, 189, 191). It is possible to identify some core microbiota in the oral cavity, esophagus, and stomach (148). Although extensive efforts have been made to cultivate representative gut microbiota in an attempt to gain a better understanding of the relationship between taxa and function (156), there are still many undescribed taxa with unknown functional roles in the gut.

As the price of sequencing decreases, it is becoming more common to use a metagenomic approach that provides information on all microbiota and potential functions (3, 70, 167, 189). This provides a means to go beyond *Bacteria* and obtain information on eukaryotic microbes (mainly fungi) and viruses. Although *Fungi*, *Archaea*, and *Viruses* in the microbiome are a part of the ‘rare biosphere’ (organisms that comprise <0.1% of the microbiome) (173), they still have a significant impact on host health.

### Fungi

*Fungi* are considered to comprise approximately 0.03% of the fecal microbiome (143); making them approximately 3,300-fold less abundant than *Bacteria*. Fungal diversity in the human gut is also lower than that of *Bacteria* (143, 166), although more taxa are being found as the number of individuals being studied using next generation sequencing is increasing (44, 126, 166, 182). In 2015, a review of 36 fungal gut microbiome studies revealed that there have been at least 267 distinct fungi identified in the human gut (181), while another study reported 221 (72). Despite the number of taxa that have been reported, most fungi are highly variable among individuals, with few appearing to be common to all.

Cultivation-based analyses have typically identified *Candida* as the most common fungal genus (166), and it is also frequently identified using non-cultivation-based methods, whereas the other taxa identified have been variable, which may be because of the analytical method used and/or subject variability. For example, 66 genera of fungi were found using pyrosequencing when 98 individuals were examined, with the genera *Saccharomyces*, *Candida*, and *Cladosporium* being the most prevalent (85). *Mucor* was common in Spanish individuals (126) and the most common fungi in 16 vegetarians were *Fusarium*, *Malassezia*, *Penicillium*, and *Aspergillus* (182). These studies suggested that some taxa, *e.g.*, *Penicillium* and *Aspergillus*, are not resident in the gut and enter through environmental sources, such as food and water, in which they are commonly found. This may account for some of the variability in taxa reported in various studies and for the increasing number of fungi being identified as more studies are being performed, even those based on cultivation (71). Under certain conditions, some fungi may flourish and become pathogenic including *Candida*, *Aspergillus*, *Fusarium*, and *Cryptococcus* (44, 84, 140, 143). More information on fungal interactions and diseases is available in a review by Wang *et al.* (204).

Despite their low abundance, fungi appear to have developed in mammalian guts along with the rest of the body from infancy (106, 169). Although there is no consensus of a core mycobiome, *Candida*, *Saccharomyces*, and *Malassezia* have been commonly reported (72). Most of the fungal species detected appear to be either transient or environmental fungi that cannot colonize the gut and are often found in a single study and/or one host only. A previous study indicated that the fungal community is unstable; only 20% of the initially identified fungi were detected again 4 months later (78). More studies on the stability of the mycobiome are needed in order to establish the ecological roles of the components of the mycobiome. Many non-bacterial organisms have been found in numerous mammalian systems, which indicates that they play an important role that has been largely overlooked and may lead to important discoveries and understanding in the coming years.

### Archaea

The most commonly reported genus of *Archaea* that has been found in the GI tract is *Methanobrevibacter* (51, 55, 66, 85, 109). Other genera that have also been detected are *Methanosphaera* (51), *Nitrososphaera*, *Thermogynomonas*, and *Thermoplasma* (85) and the new candidate species, *Methanomethylophilus alvus* (27, 131). Although *Archaea* comprise a very small proportion of the microbiota, *Methanobrevibacter* species are important contributors to methanogenesis (66). Differences in *Archaea* in microbiome samples may be due to the method used (51) and/or complex relationships with other microbiota. For example, *Methanobrevibacter* and *Nitrososphaera* were previously shown to be mutually exclusive and potentially related to carbohydrate intake (85). More studies are needed in order to clarify the interaction between *Archaea* and other microbiota groups, which may contribute to our understanding of their fitness and function (beyond methanogenesis) in the microbiome.

## Viruses

*Viruses* in the human microbiome have also been understudied and available information is limited (161); the majority of data are related primarily to disease and do not address the commensal virome (34, 40). The majority of viral reads in studies that have been performed cannot be assigned to a known group; this has contributed to the difficulties associated with assessing their roles in the GI tract (124, 160). A number of teams have made extensive efforts in order to advance human virome studies (157, 161). In the last ten years, the number of identified polyomaviruses has increased from 4 to 13 species (some that cause disease and some that do not) (47), and the accuracy of identification techniques has been improved to identify taxa at the genus level (199) and use metagenomic information for viral taxonomy (172). Viral communities are mainly comprised of bacteria-infecting phage families (~90%), while eukaryotic viruses (~10%) are in lower abundance (157, 161). Metagenomic analyses have suggested that the new bacteriophage, crAssphage associated with *Bacteroides*, is potentially common in humans (53). The greatest diversity of phages is considered to occur in infants and decreases with age, in contrast to increases in bacterial diversity (116, 117, 162). With the availability of methods to enrich viruses in samples (41), and with more metagenomic sequences and bioinformatics tools to identify viral sequences (53, 139), more information will be obtained on viral diversity and associated physiological factors in humans.

Similar to the microbiota, considerable variability appears to exist in the viral taxa found among subjects (133). Limited information is currently available on the functional roles of most viruses in the human GI tract. However, some possible functions are: to increase bacterial fitness as sources of genetic information (*e.g.*, the source of antibiotic resistance genes), to increase the immunity of bacteria or the human host, and to protect against pathogens (40, 64, 157). The general consensus is that the presence of bacteria is beneficial for viruses that are increasingly trying to evade the immune system. This relationship may also be beneficial to bacteria as viruses may be sources of potentially advantageous genes (resistance or tolerance to stress environments). Researchers are now examining the ecological and evolutionary influences of phages on bacterial ecosystems (102), and the findings obtained may provide insights into the important roles played by phages in the gut microbiome.

## The GI tract

**Fig. 1:**
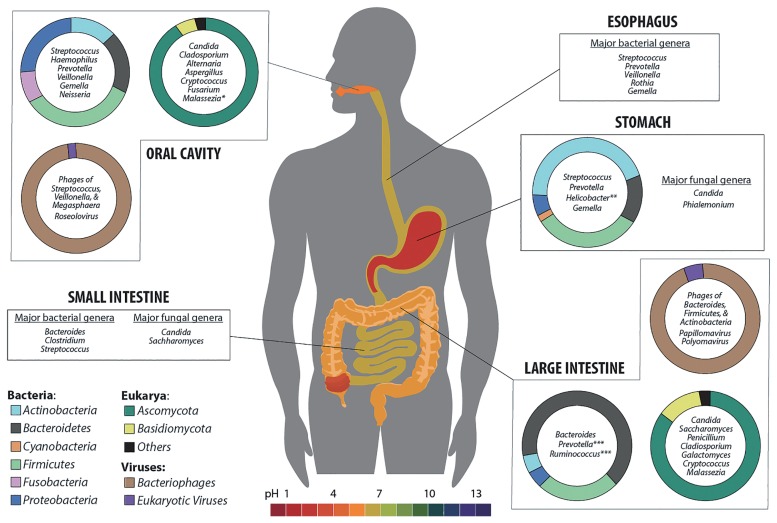
Microbiome composition of *Bacteria* (1, 5, 20, 21, 43, 147, 156, 223), *Eukarya* (52, 85, 114, 126, 182, 197), and *Viruses* (45, 134, 151, 215) among the physiological niches of the human gastrointestinal (GI) tract. Phylum level compositional data are presented where available along with the most common genera in each GI tract location. The colors on the doughnut plots correspond to the legend in the lower left corner; the GI tract is colored according to the pH scale shown at the bottom of Fig. 1. (* *Malassezia* was very abundant in one study and was not detected in another study. ** The abundance of *Helicobacter* may vary greatly between individuals. *** Proportions of these and other colon genera vary with age, diet, & geographical location.)

Many challenges are associated with studying the microbial ecology of the GI tract because it is composed of chemically and physically diverse microhabitats stretching from the esophagus to the rectum, providing a surface area of 150–200 m^2^ for colonization or transient occupation by microbes (16). The adult GI tract was initially estimated to harbor 10^14^ bacteria, 10 times more cells than the human body (16, 120); however, a more recent calculation estimates there to be 10^13^ bacteria, which is equivalent to the number of human cells (170). Lower bacterial numbers (10^3^ to 10^4^ bacteria mL^−1^ of intestinal content) are found in the upper end of the GI tract, stomach, and small intestine, in which pH is low and the transit time is short (16). The highest biodiversity (richness and evenness) of bacteria (10^10^–10^11^ bacteria g^−1^ of intestinal content) is in the colon, in which cell turnover rate is low, redox potential is low, and the transit time is long. This section highlights the different functions and associated microbiota along the human GI tract starting from the oral cavity, then the esophagus, stomach, and intestines (Fig. 1).

### The oral cavity

Activity in the mouth may have a large impact on the further digestion of food in the lower GI tract. Food is mechanically ground into small particles, typically 0.1 mm, which increases the surface area. The oral microbiome is composed of transient and commensal populations that often form biofilms on soft and hard surfaces in the mouth (8). The most up-to-date information on taxa of the oral microbiome may be found in the Human Oral Microbiome Database (HOMD, http://www.homd.org/) (50). Information in this database is limited to *Bacteria* and one *Archaea*. Cultivation-independent analyses indicate that the most common genus is *Streptococcus*, while other genera include *Neisseria*, *Gemella*, *Granulicatella*, and *Veillonella*, but not in all individuals examined (1, 91, 92, 107). The taxa present appear to be dependent on interactions between microbes within the community. For example, using a graph theory-based algorithm of an organism’s nutritional profile, the species *Streptococcus oralis* and *S. gordonii* have low metabolic complementarity and high metabolic competition, indicating they are antagonistic to each other (110). In contrast, *Porphyromonas gingivalis* was shown to have high metabolic complementarity, indicating its ability to grow symbiotically with diverse oral microbiota taxa. This computational method was tested and confirmed with growth assays, making it a viable means to assess the ability of species to inhabit the same environment. This has also been shown using an *in situ* spectral analysis of microbiota in biofilm plaques. Biofilms were shown to be composed of a number of taxa with *Corynebacterium* at the foundation (209). The other taxa are considered to play complementary roles driven by the environmental and chemical gradients formed in biofilms that control nutrient availability. These findings indicate that, despite the large number of taxa identified in oral microbiome studies, the core taxa of all microbiota may be identified in the future based on spatial locations and functional roles (10).

Similar to *Bacteria*, large variations have been noted in viruses found in the oral cavity among subjects (151). Most viruses are bacteriophages (approx. 99% of known sequences). Viral communities are reproducible across time points within a subject, suggesting that they are stable; however, the human and bacterial host significantly influence compositions (2, 151, 163). In addition to interactions among oral bacteria, many may associate with phages (57). Depending on the host range of the oral virome, this may make phages very common inhabitants of the oral cavity. Furthermore, in addition to survival within bacterial hosts, phages may also survive in the oral mucosa and contribute to host immunity (11). These are all new avenues of oral virome research that will likely be investigated in greater depth in the future.

In addition to the bacterial microbiome, two cultivation-independent studies have been conducted on oral fungi. Approximately 100 fungal species (20 genera) were detected in one study of the oral mycobiome of healthy individuals (68). Among the fungi detected, *Candida* species were the most common and abundant, while the other genera consisted of *Cladosporium*, *Aureobasidium*, *Saccharomycetales*, *Aspergillus*, *Fusarium*, and *Cryptococcus*. Most of these genera were also detected in a recent study on three subjects; however, *Malassezia*, a skin pathogen, accounted for the most sequence reads (52). Most of the other studies conducted on the oral mycobiome have focused on the role of fungi in disease (69, 136). Since the oral microbial community is directly exposed to the environment, the presence of a dynamic and transient community is expected, but warrants further study.

### Esophagus

After swallowing, food is transported down the esophagus by peristalsis to the stomach. Limited information is available on microbes inhabiting the esophagus (5, 91, 147), and this may be due to the difficulties associated with obtaining samples because biopsies have typically been used. However, a less invasive method using an esophageal string has recently been demonstrated to be a feasible alternative and yields similar findings to non-cultivation-based analyses of biopsies (60). Similar to the oral cavity, the most common genus found in the esophagus is *Streptococcus*; however, an overall comparison of the two communities has indicated that the number of taxa significantly differ between the two locations (15, 60). Among the few studies conducted on the viral and fungal microbiota of the esophagus, the focus has been on association with disease (204) and none of the pathogenic taxa inhabit healthy individuals.

### Stomach

The stomach is the first digestive organ in the body (89). It holds food and mechanically mixes it with proteolytic enzymes and gastric acids that aid in the breakdown and subsequent absorption of nutrients. The growth of many common bacteria is inhibited by these acidic conditions (pH<4), making this a unique community with the lowest number of microbes, ranging between 10^1^ and 10^3^ CFU g^−1^. In addition to digestion, the acidic conditions of the stomach are considered to have evolved as a means of protection from pathogens. This hypothesis is supported by the recent finding of a lower pH in the stomachs of scavengers and higher pH in herbivores, which are less likely to encounter pathogens in their food (13). Caution is needed when comparing the findings of various studies throughout the GI tract because gastric juice has a lower pH than the mucosal layer, resulting in differences in the microbiota present (89).

Despite the low pH, non-cultivation-based analyses on stomach biopsies revealed a more diverse microbiota than expected (5, 20, 115). Regardless of variations among subjects, there appears to be two major groups of individuals: those with and without *Helicobacter pylori* (20). There is a third subset in which *H. pylori* is present in lower proportions in some individuals that were negative using conventional testing. Microbiomes dominated by *H. pylori* had significantly greater proportions of the phylum *Proteobacteria*, of which it is a member, and lower alpha diversity (5, 20). Other common genera are *Streptococcus* and *Prevotella*, both of which are also found in the oral and esophageal communities; however, the communities at these locations appear to differ (5). Limited information is available on fungi analyzed in biopsy samples; although a cultivation study detected *Candida* species, this appeared to be associated more with disease (224). The major interaction currently studied in the stomach microbiota is with *Helicobacter* because of its association with gastritis, peptic ulcers, and gastric cancer. However, this taxon has been suggested to be beneficial for health, leading some to question whether the complete eradication of this microbe is the best option (67, 89).

In contrast, less information is available on the microbiome of stomach fluids; it appears to harbor fewer *Helicobacter* and an analysis of transcripts indicated that *Actinobacteria* are the most active phylum; however, the other major phyla, *Firmicutes*, *Bacteroidetes*, and *Proteobacteria*, are also present (197). In the same study, it also appeared to harbor novel fungi; 77.5% of the ITS reads were not identified at the phylum level or lower. *Candida* and *Phialemonium* were the only two identifiable fungal genera in all subjects tested, whereas an additional 66 genera were present in at least one of the nine subjects examined. Based on the infrequency and number of reads in this analysis, most of the taxa identified in stomach fluids appear to be transient, and those playing an active role are limited in this location.

### Intestines

After mixing in the stomach, chime slowly passes through the pyloric sphincter and enters the intestines, in which the major digestion and absorption of nutrients begin (12). Humans have a small and large intestine. The small intestine, the main location in which food digestion and absorption occurs, is further divided into three parts, the duodenum, jejunum, and ileum. The duodenum, in which food chime enters from the stomach, is directly associated with digestion and is linked to the pancreas and gallbladder. Bile salts from the gallbladder and enzymes from the pancreas enter the duodenum and mix with stomach chime in order to start the digestion process. The epithelium in the jejunum and ileum is responsible for glucose absorption into the bloodstream via glucose transporters and sodium ions. The small intestine is followed by the large intestine (colon), which has a larger diameter, but shorter length and is divided into four sections: the ascending colon (cecum), transverse colon, descending colon, and sigmoid colon (123). Water and minerals are continuously absorbed along the colon before excretion. Furthermore, complex foods that cannot be digested by the host are used as growth substrates for the colonic microbiota (25, 178).

Spatial and temporal variabilities have been noted in the microbial composition among the different intestinal structures based on their functional roles and timing of food intake (18, 129, 186). Although spatial variability exists along the intestinal tract, the bacterial microbiome at the phylum level is considered to remain fairly stable over time (43, 155); however, many factors may affect its stability (119). Undigested food and most of the microbiota are found in the lumen, the central space surrounded by the mucosal layer of the tubular intestinal structure. The main absorption of growth substrates occurs through the epithelial cells of the mucosa, which also prevents the entry of the microbiota into host cells (174). A number of important host-microbe interactions occur within the mucosa. Energy from microbially produced metabolites, such as butyrate, contributes to epithelial metabolism (97). Most of the gut is anaerobic, but there is an oxygen gradient in the mucosa that provides a competitive advantage for facultative anaerobes (174). Recent studies have also shown the importance of metabolites produced by transkingdom microbiota to host physiology (185, 187, 188). Microbiota, such as *Akkermansia mucinophila*, are commonly found residing in the mucus layer and feed on mucin (39, 48). Therefore, the effects of host interactions with the gut microbiota, particularly those in the large intestine, have a prominent impact on overall human health, including energy reabsorption and immune system development.

Due to the difficulties associated with collecting multiple samples along a healthy human GI tract in order to capture the spatial heterogeneity of microbes in this environment, most studies use fecal samples as a surrogate. However, this limits the availability of regio-specific community information on the GI tract, resulting in portions, such as the small intestine, remaining poorly characterized. The few studies conducted on the small intestine have limited subject numbers because they used biopsy samples (4, 201, 203) or ileotomy patients (108, 195, 222). The bacterial genera most commonly found among these studies were *Clostridium*, *Streptococcus*, and *Bacteroides*. The number of studies that include fungi are even more limited, with the genera *Candida* and *Saccharomyces* being the most frequently detected (108, 114). Caution is also needed when extrapolating these findings to all individuals because the health of some subjects was compromised when samples were obtained.

Bacteria in the colon account for approximately 70% of all bacteria in the human body because it is the main site for the bacterial fermentation of non-digestible food components such as soluble fiber. The small number of studies that have examined microbial communities directly in the colon suggests that the bacterial composition is similar to that found in feces (86). However, fecal communities do not represent a single colonic environment, such as the mucosa (223), but a mixture of indigenous and transient microbes from the entire GI tract. In studies in which a global view of the GI tract microbial community is of interest, fecal material represents a good surrogate and is easily obtained, allowing for multiple samples to be obtained over short and long time periods from healthy individuals. The majority of microbiome reviews have extensively covered colonic communities using feces (74, 92, 121, 148, 189); therefore, we will not describe its composition in detail. However, later in this review, the impact of diet on the microbiome composition will be discussed. Furthermore, the above sections on fungi and viruses provide information on the taxa of these groups in the intestines.

### Summary of the GI tract

The use of non-cultivation-based methods to investigate the microbiota in the GI tract has increased our knowledge of their diversity. One group that we neglected to mention in this review was *Protozoans*/*Protists*; however, recent reviews are available (79, 145). Despite representing a smaller biomass than fungi, they also appear to be important to the ecological structure of the gut microbiome. The predator-prey relationship they have with other microbiota (145) may, in some cases, lead to disease prevention (7). Difficulties are associated with elucidating the functional roles played by these various taxa at different points along the GI tract. Therefore, it is still important to obtain cultivated representatives to investigate their role and ecological significance along the GI tract. This consideration is important for all microbiota; however, it represents a larger issue for low diversity groups, such as fungi, which may not be numerically abundant, but still play a significant role (17).

## Use of animal models

Animal models have been widely adopted in human gut microbiome research (28, 98, 220) to reduce confounding experimental factors such as genetics, age, and diet, which may be more easily controlled in laboratory animals. Additionally, animal models with modified genetic backgrounds are available for investigating potential mechanisms (137). Ideally, animal models with relatively similar genetic information (217), gut structures, metabolism (142), and diets and behavior patterns (202) to humans need to be selected. Comprehensive comparisons of mice (137) and pigs (217) to humans were recently conducted in order to aid in translating information from animal models to humans. In this section, we will highlight some of their findings and compare GI tract structures and microbial community compositions. Furthermore, some advantages and limitations associated with the use of animal models in human microbiome research will be discussed.

Similarities exist in the anatomy of the GI tract between humans and most animal models (Table 1). However, differences in anatomical structures and pH at different locations along the GI tract may contribute to differences in the microbiota found in humans versus animal models (26). The human colon also has a thicker mucosal layer than those of mice and rats (137), which may have an effect on the diversity of the microbiota colonizing the colon. Human gut bacteria are dominated by two phyla: *Firmicutes* and *Bacteroidetes* (189), which also dominate the GI tract of commonly used model animals (112). However, at lower taxonomic levels, some differences have been reported in microbiome compositions in the gut between humans and animal models (Table 2). The dominant taxa reported have varied as the number of comparisons performed has increased (137, 152); therefore, the findings shown in Table 2 need to be used cautiously.

A pig gut gene catalogue of metabolic function was recently developed and compared to catalogues available for humans and mice (217). They found that 96% of the KEGG orthologs in humans were also present in pigs, whereas the overlap at the gene level was markedly lower (9.46%). However, there was a greater overlap between humans and pigs than between humans and mice. Microbial activity also differs along the GI tract, with the most relevant being fermentation occurring in the ceca of most animal models, but not in humans (137). Strengths and weaknesses are associated with the major animal models being used, and these need to be taken into consideration when conducting translational research.

### Rats

The use of rats as lab animals dates back to the 1850s. They were considered to be a good candidate for human microbiome research because the rat contains the same four dominant bacteria phyla in the GI tract (31), with *Firmicutes* (74%) and *Bacteroidetes* (23%) representing the largest proportions (221). The advantages of using rats in human microbiome research include quick reproduction, a fully sequenced genome, and easy handling and maintenance due to their relatively small size. The limitation of this model is that the diet used in rats differs from that for humans, and their behavior and living environment are also different, which will affect the gut microbiota. The diet used in rat studies is normal chow that is rich in fiber (205), and diet may rapidly alter gut microbiota diversity (46). Although most studies emphasize the impact of diet on the microbiota in the cecum and/or colon (feces), the oral cavity of rats has been used to clarify the impact of diet on the microbiome (93).

### Mice

Many of the strengths and weaknesses associated with using rats are also applicable to mice. Similar to humans, the microbiota in the GI tract of mice is dominated by *Firmicutes* (74%) and *Bacteroidetes* (23%) at the phylum level (217). However, there are differences at the genus level, and this has led to the use of “humanized” mice. This is achieved by inoculating human gut microbiota into germ-free (GF) mice (192) or mice treated with antibiotics to eliminate their gut microbiome (83). The microbiome of these mice after fecal transplants may have a composition at the phyla level that is 100% similar to humans and 88% at the genus level (137). A recent study (175) used humanized mice to test microbiome diversity after feeding with poorly accessible carbohydrates, and found a similar reduction in OTU numbers to a human study (219). However, there are also some limitations to using these animals, including the diet and environmental living conditions. Furthermore, gnotobiotic mice may not reflect the human-microbe relationship due to their weaker immune system (6).

Approximately 10 years ago, Scupham (168) showed that all four major fungal phyla, *Ascomycota*, *Basidiomycota*, *Chytridiomycota*, and *Zygomycota*, were present in the murine gut. Additionally, many genera were identified, including *Acremonium*, *Monilinia*, *Fusarium*, *Cryptococcus*, *Filobasidium*, *Scleroderma*, *Catenomyces*, *Spizellomyces*, *Neocallimastix*, *Powellomyces*, *Entophlyctis*, *Mortierella*, and *Smittium*. When comparing these studies to the human gut, it is important to note that this study indicated a more diverse fungal community than those found in humans; the eukaryotic diversity of the human gut is low (143).

### Pigs

Pigs have been used as surrogates for human microbiome research due to their highly similar genetics, physiological structures, behavior, metabolism, and immune functions to those of humans (81, 202). The greater similarities in the omnivorous diet and GI tract structure between pigs and humans are more advantageous than the murine model. The microbiome of pigs is dominated by two phyla: *Firmicutes* and *Bacteroidetes* (104); however, there are some notable differences at the genus level. The genus *Prevotella* was found to be common in two pig metagenomic studies (104, 118). Since the number of pigs used in most studies is less than humans, the pig core microbiome at the genus level may change as more pigs are studied. Another contributing factor to shaping the microbiome composition is diet. Most studies have found that the number of *Bifidobacteria* in pigs, even those on high fiber diets, is lower than that in humans (132, 218), while that of *Lactobacillus* is higher (149). In nutrition studies, humans and pigs are both dependent on the quality of the nutrient load; however, the pig cecum has a larger capacity to ferment indigestible compounds than the human cecum (54). The microbiota composition in pigs may differ from that in humans due in part to differences in diet (81). Similar to mice, humanized GF pigs have been developed and the microbiome after human fecal transplantation more closely resembles that of the donor than conventional pigs (144). However, the same disadvantages associated with using GF mice are also true for GF pigs.

The genome of pigs may be mutated to study human diseases; this is typically performed using miniature pigs such as those from the Ossabaw and Gottingen islands (146). Genetic mutations for metabolic syndrome and insulin resistance have successfully been performed using Ossabaw pigs to study human diseases such as type 2 diabetes (14, 177) and obesity (101). The ratio of *Firmicutes* to *Bacteroidetes* is higher in obese Ossabaw pigs than in lean pigs (146), similar to some obese humans (111, 190). This finding suggests that Ossabaw pigs are a good model for researching the role of the microbiota in human obesity. However, disadvantages are associated with using miniature pigs, mainly the higher cost for maintenance and longer reproductive period than rodents (146).

Although more extensive efforts have been made to investigate fungi in pigs than in other animal models, many of these studies were cultivation-based or for use as probiotics. Fungi in pigs have been recently studied using a non-cultivation approach and up to 17 species of yeast (belonging to the genera *Kazachstania*, *Galactomyces*, *Candida*, *Issatchenkia*, *Pichia*, *Rhodotorula*, and *Trichosporon*) were common in the gut (194). The number of studies on viruses is limited, but the composition appears to be highly variable among samples (164, 171) and affected by disease (24). These groups need to be examined in more detail in order to establish whether pigs are good models for use in understanding fungi and viruses in humans.

### Animal model summary

The convenience and cost of using animal models for human research are appealing. However, researchers need be very careful when selecting animal models appropriate for their objectives, particularly when the objective is to directly extrapolate findings from animals to humans, due to the significant differences in GI tract physiology and microbiome composition (65, 137, 217).

## Diet in health

Many studies have found that diet is one of the main factors shaping the composition of gut microbial populations. Dietary approaches, such as the ingestion of non-digestible carbohydrates (prebiotics) and fermented food products containing live cultures (probiotics), have been suggested to confer health benefits by enhancing the growth of beneficial intestinal bacteria (100, 158). As described earlier, the microbiota may break down food components, such as non-digestible carbohydrates, which are indigestible by the host in order to aid in maximizing available nutrients (9) and produce metabolites that contribute to host health. Probiotics have been used as a means to replenish the gut with “beneficial” microbiota after antibiotic treatments or to treat diseases (82, 159). This section will highlight some studies that demonstrated the health benefits of prebiotics and probiotics and possible roles played by the microbiota.

### Dietary prebiotics and probiotics

Non-digestible and fermentable food components are often consumed as prebiotics to selectively stimulate the growth and/or activity of endogenous colonic bacteria that may be beneficial to host health. The increased consumption of prebiotics often correlates with enhancements in certain bacterial genera (a common example is *Bifidobacterium* sp.); however, the reason they are beneficial remains unclear (208). Challenges are associated with elucidating the role being played by specific bacterial phylotypes because many of their processes are interactive (207). For example, SCFA produced by bacterial fermentation may lower intestinal pH, thereby increasing the solubility of essential minerals, such as calcium, iron, and magnesium, and consequently enhancing their absorption and improving health. Metabolites produced by microbes may also play an important role in cellular differentiation and proliferation in the colonic mucosa by inducing apoptosis and may confer protection against colitis and colorectal cancer by modulating oncogene expression. These functions do not appear to be performed by a single species; a number of different species may be acting independently or in combination. Research is leading to an understanding of microbial community structure and composition dynamics with respect to diet aids in establishing testable hypotheses for future research in health and beneficial microbes (32). Most research has been performed on the influence of beneficial intestinal bacteria such as *Bifidobacterium* spp. and *Lactobacillus* spp. on host health monitored using a cultivation approach. Cultivation-independent approaches have now become more popular, leading to the identification of new beneficial microbiota taxa and their potential functional roles in the gut as they relate to diet.

Dietary fibers and oligosaccharides are carbohydrate ingredients that vary in composition and structure, but are considered to be non-digestible because of the lack of appropriate intestinal enzymes to hydrolyze them or structural hindrances that prevent enzyme access in the gut. Although bacteria in the lower gut may ferment these carbohydrates, the rate and degree of fermentation vary with the polysaccharide (80). The range of fermentation in the colon for various fibers is broad, from approximately 5% for cellulose to nearly 100% for pectin (42). The resulting SCFA, including butyrate and propionate, are considered to reduce pH and solubilize minerals, thereby improving their absorption and subsequent utilization. Inulin, a long chain fructooligosaccharide (FOS) often obtained from chicory root, and FOS from other sources are the fibers that have been studied in the most detail (206). Several novel fibers have been tested in an *in vitro* large intestine model for their effects on the microbial stimulation and production of SCFA (122). All these novel fibers stimulated the growth of beneficial *Bifidobacteria* and some *Lactobacillus* species along with increases in SCFA production. Only a few studies have examined the effects of fibers and resistant starches on the human microbiome (87, 127, 198, 210, 211). A soluble corn fiber product has been demonstrated to increase Ca absorption in a number of different studies (210, 211). More benefits to human health may be attributed to the consumption of prebiotics and fermentation by the gut microbiome.

The number of studies that include diet effects on *Archaea*, *Fungi*, and/or *Viruses* are limited; however, some examples are included herein. Examinations of *Archaea*, *Fungi*, and *Bacteria* correlations in response to diet revealed a syntrophic model involving *Candida*, *Prevotella*, *Ruminococcus*, and *Methanobrevibacter* (85). *Candida* was considered to break down carbohydrates into metabolites used by *Prevotella* and *Ruminococcus* that produce CO_2_ for *Methanobrevibacter* (85). However, shifts in carbon sources or breaking down starches via amylases from the human mouth may alter this relationship because *Prevotella* may no longer be dependent on *Candida*. This is a good example of how *Archaea*, which represent a very small portion of the microbiome, are a key contributor to methanogenesis and waste decomposition. The absence of *Archaea* may have severe effects on the surrounding community as hydrogen, glucose metabolites, and other carbon sources accumulate. Other organisms will eventually fill this niche, but may diminish or accumulate new metabolites that ultimately shift the surrounding community based on their fitness for using these substrates.

A recent study investigated rapid changes in the microbiome composition when diets were either high in animal-based or plant-based fat and protein (46). The fungus *Candida* was found to increase in subjects placed on a plant-based diet, whereas *Penicillium* increased on animal-based diets. The most commonly found fungi in vegetarians were *Fusarium*, *Malassezia*, *Penicillium*, *Aspergillus*, and *Candida* (182). Caution is needed when interpreting findings because some of these fungi may be found on food prior to ingestion (46, 78, 182)

Phages assembled in the gut may also be modified by diet. A recent study examined changes in the fecal viral community over an 8-d period in six subjects supplied different diets (134). Shotgun sequencing of virus-like particles revealed that interpersonal differences in the virome were the largest source of variations in this study. However, the virome of subjects whose diets were changed differed more than in those who maintained their normal diet. Although this is only one study with a few human subjects, studies using a mouse model and different dietary fats support these findings (88, 99). Collectively, these findings indicate that diet plays a key role in shaping the gut virome, and further research is needed in order to investigate interactions between diet and the virome.

## Summary

Advances have been made in the last decade in our understanding of the role of the GI tract microbiome in human health. This review has highlighted changes and differences in the microbiome along the GI tract that are due to changes in physical, chemical, and biological interactions. Although extensive research has been conducted on *Bacteria* in fecal samples, the main kingdom inhabiting the gut, our knowledge is still insufficient, particularly in other regions of the GI tract. Furthermore, other groups (*Archaea*, *Fungi*, and *Viruses*) have not yet been investigated in adequate detail, demonstrating a real void in knowledge. This highlights that the basic ecology of microbiomes is important for gaining a greater understanding to improve human health and decrease disease. In order to achieve this goal, it is important to include all microbiota in studies and remain cognizant of the limitations associated with understanding the entire GI tract of humans despite challenges in sampling and cultivation. Furthermore, the use of appropriate animal models in mechanistic studies requires careful consideration.

## Figures and Tables

**Table 1:** Comparison of the anatomy of the intestinal tract in humans and animal models

	Human	Mouse	Rat	Pig
Stomach	Four regions: cardia, fundus, body, and pylorus	Three regions: forestomach, body, and pylorus	Three regions: forestomach, body, and pylorus	Four regions: esophagus, cardia, fundus, and pylorus
	pH 1.5 to 3.5	pH 3.0 to 4.0	pH 3.0 to 4.0	pH 1.5 to 2.5
Small intestine	5.5–6.4 m in length	350 mm in length	1,485 mm in length	1.2–2.1 m in length
	pH 6.4 to 7.3	pH 4.7 to 5.2	pH 5.0 to 6.1	pH 6.1 to 6.7
Cecum	Smaller than the colon	Larger than the colon	Larger than the colon	Smaller than the colon
	No fermentation	Main fermentation	Main fermentation	Some fermentation
	pH 5.7	pH 4.4 to 4.6	pH 5.9 to 6.6	pH 6.0 to 6.4
Appendix	Present	Absent	Absent	Absent
Colon	Divided into the ascending, transcending, and descending colon	Not divided	Not divided	Divided into the ascending, transcending, and descending colon
	Main fermentation	No fermentation	No fermentation	Main fermentation
	Thick mucosa	Thinner mucosa	Thinner mucosa	Thick mucosa
	pH 6.7	pH 4.4 to 5.0	pH 5.5 to 6.2	pH 6.1 to 6.6

Adapted from (59, 96, 128, 130, 137, 196)

**Table 2:** Major taxa of the gut microbiota in humans and animal models

	Human	Mouse	Rat	Pig
*Bacteria*	*Firmicutes*	*Firmicutes*	*Firmicutes*	*Firmicutes*
	*Bacteroidetes*	*Bacteroidetes*	*Bacteroidetes*	*Bacteroidetes*
	*Actinobacteria*			
	*Proteobacteria*			
*Archaea*	*Methanobrevibacter*	*Methanobrevibacter*	*Methanobrevibacter*	*Methanomicrobia*,
	*Nitrososphaera*			*Methanosphaera*
*Viruses*	*Herpesviridae*	Variable	Variable	*Picornaviridae*
	*Papillomaviridae*			*Astroviridae*
	*Polyomaviridae*			*Coronaviridae*
	*Adenoviridae*			*Caliciviridae*
*Eukarya*	*Candida*	*Ascomycota*	*Ascomycota*	*Kazachstania*
	*Malassezia*	*Basidiomycota*	*Basidiomycota*	*Candida*
	*Saccharomyces*	*Chytridiomycota*	*Chytridiomycota*	*Galactomyces*
	*Cladosporium*	*Zygomycota*	*Zygomycota*	*Issatchenkia*

Adapted from (85, 103, 105, 112, 125, 137, 153, 154, 171, 179, 193, 194, 215, 216, 221)
